# Tumor budding in pre-neoadjuvant biopsy and post-neoadjuvant resection specimens is associated with poor prognosis in intrahepatic cholangiocarcinoma—a cohort study of 147 cases by modified ITBCC criteria

**DOI:** 10.1007/s00428-024-03937-y

**Published:** 2024-10-10

**Authors:** Gaohua Wu, Rongkui Luo, Qianhui Xu, Liuxiao Yang, Hongping Xia, Valerie Chew, Ye Xin Koh, Kenneth Tou En Chang, Jian Zhou, Jia Fan, Qiang Gao, Ruoyu Shi, Kai Zhu

**Affiliations:** 1 Department of Liver Surgery and Transplantation, Liver Cancer Institute, Zhongshan Hospital, Fudan University, 180 Fenglin Road, Shanghai, P.R. China 20032; 2 Department of Pathology, Zhongshan Hospital, Fudan University, Shanghai, China; 3 Zhongda Hospital, School of Medicine & Advanced Institute for Life and Health, Southeast University, Nanjing, China; 4 SingHealth-DukeNUS Academic Medical Centre, Translational Immunology Institute (TII), Singapore, Singapore; 5 Department of Hepatopancreatobiliary and Transplant Surgery, Singapore General Hospital and National Cancer Centre Singapore, Singapore, Singapore; 6 Department of Pathology and Laboratory Medicine, Kandang Kerbau Women’s and Children’s Hospital, 100 Bukit Timah Road, Singapore, 229899 Singapore

**Keywords:** Intrahepatic cholangiocarcinoma, Lymphovascular invasion, Perineural invasion

## Abstract

Tumor budding (TB) has been associated with poor survival in a variety of cancers including intrahepatic cholangiocarcinoma (iCCA). As tumor histomorphological features are significantly altered after neoadjuvant therapy (NAT), our study aims to assess the prognostic significance of TB in iCCA patients before and after NAT, by the modified International Tumor Budding Consensus Conference (ITBCC) criteria. 147 NAT-treated iCCA cases were included in this study. In biopsy specimens obtained before NAT, the TB-positive subgroup had lower overall survival (OS) in univariate analysis (*P* = 0.010). In resection specimens obtained after NAT, the TB-positive subgroup had reduced OS (*P* = 0.002) and recurrence-free survival (RFS) (*P* = 0.013) in univariate analysis. In multivariate analysis including TNM stage, lymphovascular invasion and perineural invasion, TB-positive in post-NAT resection was also found as an independent prognostic factor for both OS and RFS (OS, HR, 3.005; 95% CI, 1.333–6.775, *P* = 0.008; RFS, HR, 1.748; 95% CI, 1.085–2.816, *P* = 0.022). In conclusion, assessing the presence of TB by modified ITBCC criteria provides robust prognostic information in the NAT setting of iCCA patients and can be considered to be included in routine pathological reporting.

**Supplementary Information:**

The online version contains supplementary material available at 10.1007/s00428-024-03937-y.

## Introduction

Intrahepatic cholangiocarcinoma (iCCA), the second most lethal primary liver cancer after hepatocellular carcinoma, is expected to experience a ten-fold increase in global incidence over the next two to three decades [1]. iCCA patients have a 5-year survival rate of less than 10% due to late diagnosis, aggressive biological behavior, and limited options for effective treatment [2, 3]. Surgical resection is currently the only curative-intent treatment for early- to intermediate-stage resectable iCCA patients. Nevertheless, the 3-year recurrence rate is reported to be as high as 80%, which negatively impacts the outcome of surgery [4, 5].

In the past, when conventional chemotherapy was the mainstream treatment modality in late-stage iCCA, limited evidence showed the benefit of neoadjuvant therapy (NAT) [6, 7]. In the era of immunotherapy, the combination of immune checkpoint inhibitors with chemotherapy and/or targeted shows promising results in treating advanced iCCA by downstaging the tumor, increasing the rate of complete resection, and reducing the rate of local recurrence [8–11]. The phase III randomized clinical trial TOPAZ-1 (NCT03875235) has shown significant improvement in treating advanced iCCA patients. The regimen used in this trial (durvalumab plus gemcitabine and cisplatin) has since been approved by the FDA for locally advanced or metastatic biliary tract cancer [12, 13]. Another phase III clinical trial (NCT04003636) with a larger patient cohort, involving pembrolizumab plus gemcitabine and cisplatin, also demonstrated that advanced iCCA patients can benefit from systematic immunotherapy [10]. Both results indicate that more iCCA patients can benefit from systematic immunotherapy, which also enlightens the future for borderline resectable iCCA patients [12, 14–16]. Evidence from clinical practice also reveals that initially unresectable or borderline resectable iCCA patients can benefit from combined immunotherapy by downstaging the tumor and eventually undergoing surgical resection [17–19]. Nevertheless, the effectiveness of combined immunotherapy varies significantly among iCCA patients [20, 21]. Clinicopathological parameters and biomarkers that could stratify patients into different prognostic and potentially therapeutic subgroups would be highly beneficial. Conventional pathological parameters, such as tumor size and grade, are known to be profoundly altered after treatment [22, 23]. To date, reliable clinicopathological parameters that can predict the survival of post-NAT iCCA patients are not well reported in the literature.

Tumor budding (TB) is a histomorphological parameter defined as the presence of single cell or tumor cell clusters comprising fewer than five cells within or at the invasive front of the tumor [24, 25]. High TB counts have been reported to indicate poor prognosis in various types of cancers, including recently published results in iCCA [26–30]. Notably, the International Tumor Budding Consensus Conference (ITBCC) criteria have been recommended and adopted into the routine pathology report protocol for colorectal cancer resection specimens [31], after its prognostic value was validated in large prospective and population-based cohorts [32–34]. On the other hand, high TB counts have also been reported as an adverse prognostic factor in biopsy specimens from different types of cancers [35, 36]. Increasing evidence from post-NAT studies investigating TB in rectal [37], esophageal [38], gastric [39] cancers, and perihilar cholangiocarcinoma [40] suggests that even after NAT, where conventional histopathological grading is usually omitted, the prognostic power of TB is retained.

In this study, we evaluated tumor budding in pre-NAT biopsy specimens and subsequent surgically resected iCCA specimens from patients who received NAT, to determine its prognostic significance.

## Materials and methods

### Patient cohort and follow-up

This study included a total of 147 iCCA patients who received NAT and underwent surgery in the Department of Liver Surgery and Transplantation, Liver Cancer Institute, Zhongshan Hospital, Fudan University between January 2019 and October 2023. All patients had histologically or clinically confirmed iCCA and were considered initially unsuitable for surgery by our multidisciplinary team (MDT). MDT suggested patients to receive 6 to 8 cycles of treatment prior to a second MDT discussion. The treatment regimen was gemcitabine, oxaliplatin, lenvatinib, and anti-PD1 antibody. A surgery was performed after MDT confirmed patients were suitable for resection. The clinical and follow-up information, including age at diagnosis, gender, date of surgery, and the date and site of recurrence/metastasis, and/or death, when applicable, were obtained from medical records and a follow-up database which was updated every 3 months. Local disease recurrence and distant metastasis were determined based on clinical and radiographic evaluation during the follow-up visits.

The study was approved by the Zhongshan Hospital Research Ethics Committee Research Ethics Committee and complied with all ethical regulations(Approval ID: B2023-321, IRB number: IRB00014212).

### Assessment of histological samples

Hematoxylin and eosin (HE)-stained slides, from all biopsy and surgical resection samples, were retrieved from the archive. They were scanned and digitized using the KF-PRO-400 Digital Slide Scanner (KFBIO CO., Yuyao, China). Two experienced histopathologists (RY Shi and RK Luo) evaluated all the slides and graded TB independently, blind to the follow-up data. Discrepant cases were discussed with a third senior pathologist (KTE Chang) together to reach the final counting consensus. TB grade in 6 biopsy cases and 10 resection cases were found discordant and eventually obtained a consensus on budding status.

In this study, we used the definition of tumor budding from the 2016 ITBCC criteria: A tumor bud is defined as a single tumor cell or a cell cluster of up to four tumor cells [37] and assessed TB counts in all available slides with tumor and counted the TB in a “hotspot” field measuring 0.785 mm^2^ (objective magnification, × 20).

For the resection specimens, we initially graded TB count as low (0 to 4 buds), intermediate (5 to 9 buds), or high (≥ 10 buds) based on ITBCC criteria and subsequently modified intermediate and high grades as TB-positive subgroup and low TB grade as TB-negative subgroup. We assessed TB in whole tumor areas in the entire tumor bed rather than at the invasive front because treatment-related changes such as necrosis, fibrosis, and hemorrhage made the determination of the front frequently difficult.

For biopsy specimens, we used the receiver operating characteristic (ROC) curve to determine the best cut-off value of TB counts to predict survival. A TB-positive subgroup is defined as having TB counts more than the cut-off value.

Tumor regression scores (TRS) were assessed according to the grading scheme recommended by the College of American Pathologists (CAP) for pancreatic cancer [41].

### Statistical analysis

Statistical analysis was performed using the SPSS 27.0 software package (IBM Corp., Armonk, N.Y., USA). The chi-square test or the Fisher exact test was employed to compare differences between two independent subgroups. RFS and OS curves for the subgroups were generated by GraphPad Prism (GraphPad Software, San Diego, California, USA) using the Kaplan–Meier method and compared using the log-rank test. The univariate and multivariate analyses were performed using Cox regression analysis. The correlation of tumor buds in pre- and post-NAT specimens were defined by the Spearman correlation test. A 2-sided significance level of 0.05 was used for all statistical analyses.

## Results

**Fig. 1:**
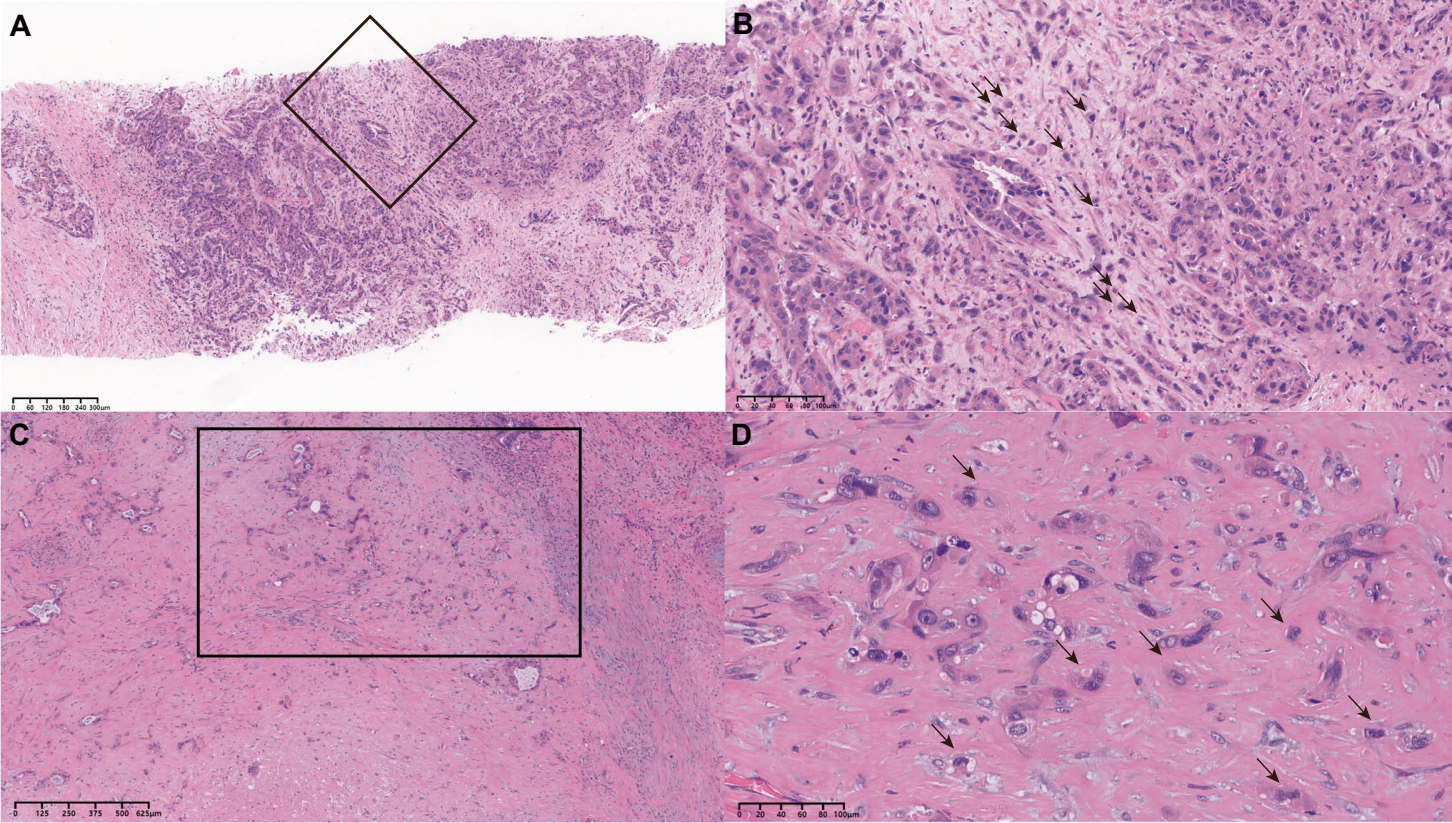
Representative HE images of tumor buds. **A** Pre-NAT biopsy specimens in low power view. **B** High power view of the rectangular area in A demonstrating TB can be readily identified in the biopsy. **C** Post-NAT resection specimens in low power view, residual tumor cells are present in the background of treatment-related changes such as fibrosis and foamy histocyte aggregation. **D** High power view of the rectangular area in C demonstrating TB can be readily identified, despite treatment-related changes. HE indicates hematoxylin–eosin; NAT, neoadjuvant therapy; TB, tumor budding

A total of 147 cases were included in our study, comprising 95 cases of biopsy material obtained from patients before NAT and 139 cases of post-NAT resection with viable tumor cells for TB assessment. All surgical margins were negative, with a range of 0.1 to 5.0 cm. Eight cases were not included in post-treatment analysis, because 5 cases showed complete tumor necrosis and 3 cases of resection specimen slides were not available for us. A total of 87 cases had paired pre-NAT biopsy and post-NAT resection specimens for TB assessment. The median follow-up time was 29 months, during this follow-up 79 patients (53.7%) developed disease progression and 32 patients (21.8%) died. TB counts could be readily identified in both biopsy and resection specimens (Fig. 1). cTNM stage was confirmed before NAT. TRS did not show significant association with TB grade (*P* = 0.395) (Table 1). TRS grade 3 did not show significant association with either OS (*P* = 0.870) or RFS (*P* = 0.205) in the univariate analysis (Tables 2,3), as well as on Kaplan–Meier curves for either OS (*P* = 0.382) or RFS (*P* = 0.052) (Supplemental Fig. 1).

**Table 1:** Associations between tumor budding and clinicopathologic features of 139 post-NAT resection iCCA patients

	Surgery		
Parameter	TB-negative (%)	TB-positive (%)	*P*
Age			0.810
< 65	47 (67.1)	45 (65.2)	
≥ 65	23 (32.9)	24 (34.8)	
Gender			0.633
Male	45 (64.3)	47 (68.1)	
Female	25 (35.7)	22 (31.9)	
HBV infection			0.077
Negative	43 (61.4)	52 (75.4)	
Positive	27 (38.6)	17 (24.6)	
CEA (ng/mL)			0.152
< 5	62 (88.6)	55 (79.7)	
≥ 5	8 (11.4)	14 (20.3)	
CA19-9 (U/mL)			0.184
< 37	51 (72.9)	43 (62.3)	
≥ 37	19 (27.1)	26 (37.7)	
cTNM			0.785
I	6 (8.6)	6 (8.7)	
II	5 (7.1)	7 (10.1)	
III	56 (80.0)	51 (73.9)	
IV	3 (4.3)	5 (7.2)	
ypTNM			0.751
I	12 (17.1)	15 (21.7)	
II	4 (5.7)	4 (5.8)	
III	51 (72.9)	45 (65.2)	
IV	3 (4.3)	5 (7.2)	
Histologic grade			0.813
I	40 (57.1)	40 (58	
II	27 (38.6)	28 (40	
III	3 (4.3)	1 (1.4)	
Tumor size (median = 50) (mm)			0.943
< 50	29 (41.4)	29 (42.0)	
≥ 50	41 (58.6)	40 (58.0)	
Tumor number			0.393
1	49 (70.0)	44 (63.8)	
2	9 (12.9)	9 (13.0)	
≥ 3	12 (17.1)	16 (23.2)	
Histologic type^a^			0.055
Large duct type	21 (37.5)	11 (20.8)	
Small duct type	35 (62.5)	42 (79.2)	
Hepatic steatosis			0.805
Negative	54 (77.1)	52 (75.4)	
Positive	16 (22.9)	17 (24.6)	
Liver cirrhosis			0.637
Negative	60 (85.7)	61 (88.4)	
Positive	10 (14.3)	8 (11.6)	
Vascular invasion			0.716
Negative	65 (92.9)	66 (95.7)	
Positive	5 (7.1)	3 (4.3)	
Lymphovascular invasion			0.437
Negative	58 (82.9)	53 (76.8)	
Positive	12 s(17.1)	16 (23.2)	
Perineural invasion			0.034*
Negative	60 (85.7)	50 (72.5)	
Positive	10 (14.3)	19 (27.5)	
TRS grade			0.395
Grade 1	5 (7.1)	2 (2.9)	
Grade 2	48 (68.6)	53 (76.8)	
Grade 3	17 (24.3)	14 (20.3)	

^a^In 30 cases, histologic type is difficult to be assessed due to severe morphological alteration after neoadjuvant therapy. *iCCA* indicates intrahepatic cholangiocarcinoma, *TB* tumor budding, *HBV* hepatitis B virus, *TRS* tumor regression score

**Table 2:** Univariate and multivariate analyses of factors associated with overall survival of 139 post-NAT resection iCCA patients

	Univariate		Multivariate	
Parameter	HR	*P*	HR	*P*
Age (≥ 65)	1.782 (0.889–3.570)	0.103		
Gender (male)	1.035 (0.499–2.148)	0.927		
HBV infection status (positive)	0.869 (0.390–1.936)	0.731		
CEA (≥ 5 ng/mL)	1.796 (0.778–4.149)	0.170		
CA19-9 (≥ 37 U/mL)	1.986 (0.993–3.974)	0.052		
cTNM3/4	1.495 (0.525–4.254)	0.451		
ypTNM3/4	2.122 (0.745–6.040)	0.159		
Histologic grade (poor)	1.779 (0.883–3.582)	0.107		
Tumor size (≥ 50 mm)	1.507 (0.713–3.183)	0.282		
Tumor number (≥ 3)	1.337 (0.600–2.979)	0.478		
Histologic type (large duct type)	1.586 (0.657–3.830)	0.305		
Hepatic steatosis	1.223 (0.565–2.647)	0.610		
Liver cirrhosis	0.410 (0.098–1.716)	0.222		
Vascular invasion	0.458 (0.063–3.359)	0.443		
Lymphovascular invasion	2.860 (1.395–5.864)	0.004*	1.906 (0.907–4.005)	0.089
Perineural invasion	2.742 (1.345–5.588)	0.006*	1.512 (0.705–3.242)	0.288
TB subgroup (TB-positive)	3.491 (1.611–7.567)	0.002*	3.005 (1.333–6.775)	0.008*
TRS grade 3	1.054 (0.562–1.974)	0.870		

*NAT* indicates neoadjuvant therapy, *iCCA* intrahepatic cholangiocarcinoma, *TB* tumor budding, *HBV* hepatitis B virus, *TRS* tumor regression score

**Table 3:** Univariate and multivariate analyses of factors associated with recurrence-free survival of 139 post-NAT resection iCCA patients

	Univariate		Multivariate	
Parameter	HR	*P*	HR	*P*
Age (≥ 65)	0.991 (0.617–1.593)	0.972		
Gender (male)	1.205 (0.746–1.946)	0.446		
HBV infection status (positive)	1.069 (0.654–1.746)	0.791		
CEA (≥ 5 ng/mL)	0.991 (0.524–1.874)	0.977		
CA19-9 (≥ 37 U/mL)	1.400 (0.883–2.219)	0.152		
cTNM3/4	3.193 (1.466–6.957)	0.003*	0.734 (0.118–4.567)	0.740
ypTNM3/4	3.769 (1.881–7.551)	< 0.001*	5.439 (1.063–27.835)	0.042*
Histologic grade (poor)	1.268 (0.808–1.988)	0.302		
Tumor size (≥ 50 mm)	1.714 (1.077–2.728)	0.023*	1.704 (1.018–2.853)	0.043*
Tumor number (≥ 3)	2.131 (1.280–3.550)	0.004*	1.364 (0.766–2.431)	0.292
Histologic type (large duct type)	0.993 (0.563–1.755)	0.982		
Hepatic steatosis	0.839 (0.493–1.426)	0.516		
Liver cirrhosis	0.731 (0.351–1.524)	0.404		
Vascular invasion	0.958 (0.349–2.626)	0.933		
Lymphovascular invasion	2.599 (1.580–4.275)	< 0.001*	1.801 (1.027–3.156)	0.040*
Perineural invasion	2.250 (1.370–3.694)	0.002*	1.537 (0.888–2.661)	0.125
TB subgroup (TB-positive)	1.772 (1.126–2.789)	0.013*	1.748 (1.085–2.816)	0.022*
TRS grade 3	1.346 (0.850–2.131)	0.205		

*NAT* indicates neoadjuvant therapy, *iCCA* intrahepatic cholangiocarcinoma, *HBV* hepatitis B virus, *TB* tumor budding, *TRS* tumor regression score

### Prognostic significance of tumor budding in pre-neoadjuvant biopsy

**Fig. 2:**
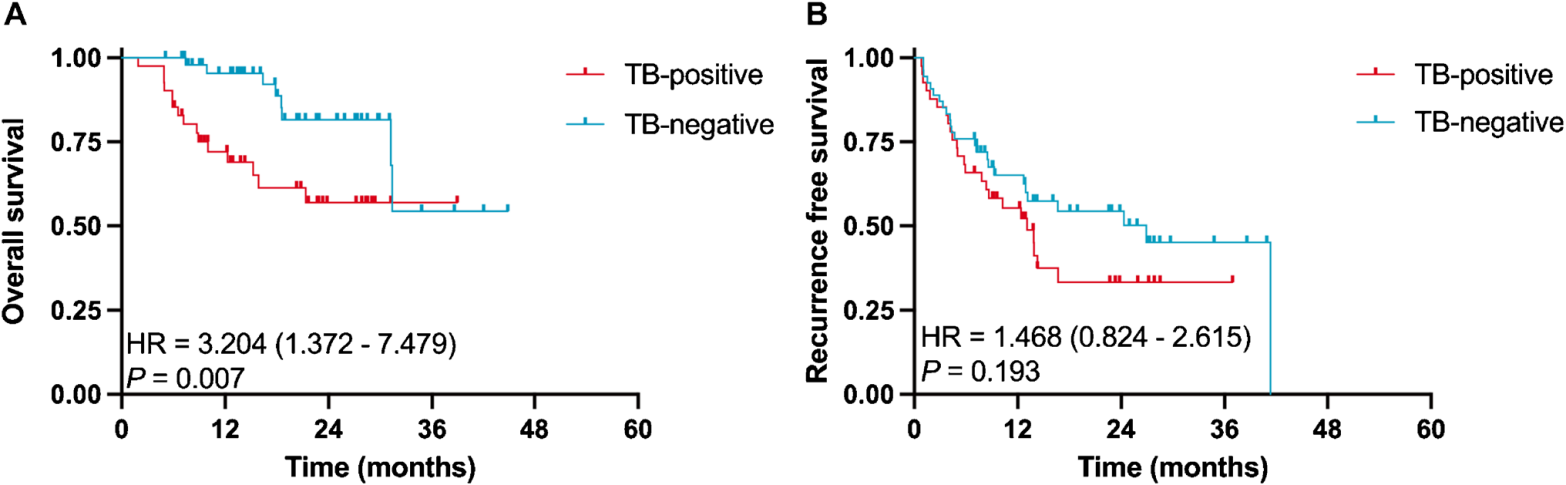
Survival analysis of pre-NAT biopsied iCCA using modified ITBCC criteria. **A** The OS of TB–positive/TB–negative cases using modified ITBCC criteria. **B** The RFS of TB–positive/TB–negative cases using modified ITBCC criteria. NAT indicates neoadjuvant therapy; iCCA, intrahepatic cholangiocarcinoma; ITBCC, International Tumor Budding Consensus Conference; OS, overall survival; TB, tumor budding; RFS, recurrence-free survival

In biopsy specimens before NAT, the mean number of biopsy cores per case was 1.96 and the median biopsy core length was 1.50 cm. TB was present in 85 cases with a mean count of 3.7. We used ROC analysis with OS as an endpoint to determine the optimal cut-off number of TB. The optimal number of tumor buds in biopsy specimens was 3 (sensitivity = 0.652, specificity = 0.639) per field. Therefore, the threshold was set at 3 buds. At this threshold, the AUC was 0.671 (*P* = 0.014) (Supplemental Fig. 2). Forty-one cases (43.2%) were assigned to the TB-positive subgroup. Supplemental Table 1 summarizes the clinicopathological characteristics of the 95 biopsy cases. A significant association was observed between the TB-positive subgroup and serum CA19-9 level (*P* = 0.006). On Kaplan–Meier curves, we observed significant differences between TB-positive and TB-negative subgroups in OS (1-year OS 68.9% vs 95.4%, 3-year OS 56.9% vs 54.4%, *P* = 0.007) (Fig. 2), indicating a better outcome in TB-negative patients. However, such difference did not achieve statistical significance in RFS (*P* = 0.193).

In the univariate analysis, the clinicopathologic features associated with shorter OS include tumor number (≥ 3) (*P* = 0.041) and TB-positive subgroup (*P* = 0.010) (Supplemental Table 2), whereas advanced cTNM stage and tumor number (≥ 3) (*P* = 0.013) were related with shorter RFS. The multivariate analysis demonstrated that tumor number (≥ 3) (hazard ratio (HR), 2.510; 95% CI, 1.042–6.046, *P* = 0.040) and TB-positive subgroup (HR, 2.806; 95% CI, 1.113–7.077, *P* = 0.029) were correlated with shorter OS (Supplemental Table 2), while tumor number (≥ 3) (HR, 2.840; 95% CI, 1.437–5.613, *P* = 0.003) indicated a shorter RFS (Supplemental Table 3).

### Prognostic significance of tumor budding in post-neoadjuvant resection

**Fig. 3:**
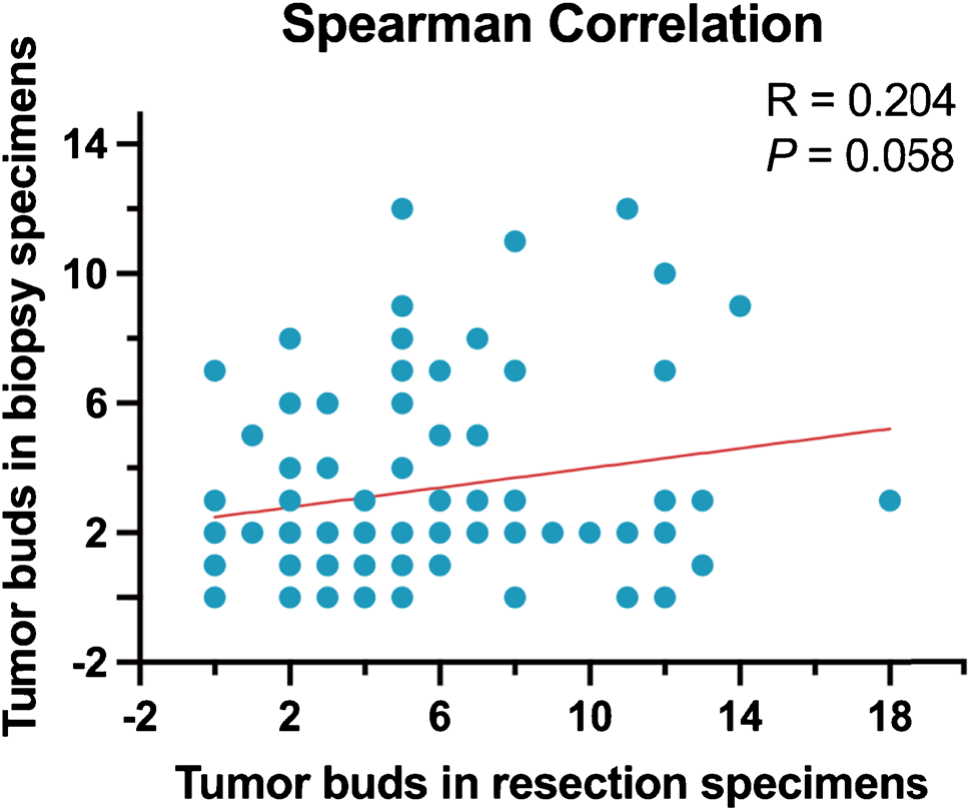
Scatterplot of tumor buds in resection specimens with tumor buds in biopsy specimens (*n* = 87) (Spearman correlation)

**Fig. 4:**
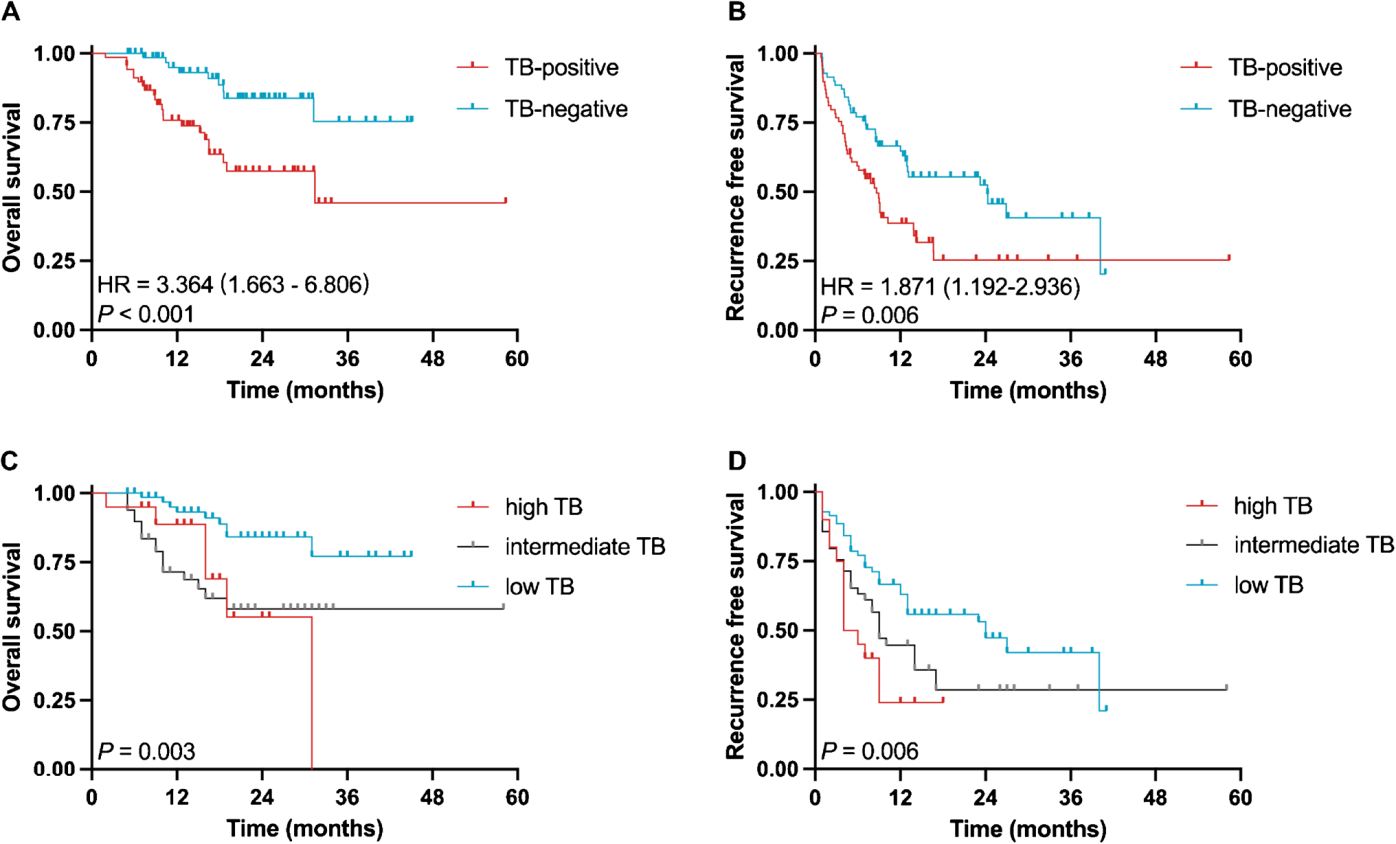
Survival analysis of post-NAT resected iCCA using modified or standard ITBCC criteria. **A** The OS of TB–positive/TB–negative cases using modified ITBCC criteria. **B** The RFS of TB–positive/TB–negative cases using modified ITBCC criteria. **C** The OS of high TB/intermediate TB/low TB cases using standard ITBCC criteria. **D** The RFS of high TB/intermediate TB/low TB cases using standard ITBCC criteria. NAT indicates neoadjuvant therapy; iCCA, intrahepatic cholangiocarcinoma; ITBCC, International Tumor Budding Consensus Conference; OS, overall survival; TB, tumor budding; RFS, recurrence-free survival

In post-NAT resection specimens, TB was present in 121 cases with a mean count of 5.8, and 69 cases (49.6%) were assigned to the TB-positive group. The clinicopathological characteristics of the 139 resection cases are summarized in Table 1. We observed a weak correlation between the TB-positive subgroup in pre-NAT biopsy and the TB-positive subgroup in post-NAT resection through Spearman correlation analysis (*P* = 0.051, *R* = 0.204) (Fig. 3). Thirty patients (34.9%) had a TB status switch before and after NAT. The only histological feature associated with the TB-positive subgroup was perineural invasion (*P* = 0.034). Through Kaplan–Meier survival analyses, we found the prognostic value of standard ITBCC criteria was weak in post-NAT iCCA, especially between the high TB grade and the intermediate TB grade in both OS and RFS (Supplemental Fig. 3). However, by using modified ITBCC criteria, we observed that patients in the TB-positive subgroup had significantly shorter OS (1-year OS 73.9% vs 93.1%, 3-year OS 46.0% vs 75.4%, *P* < 0.001) and RFS (1-year RFS 40.4% vs 64.8%, 3-year RFS 26.5% vs 40.6%, *P* = 0.006) compared to those in the TB-negative subgroup (Fig. 4).

In the univariate analysis, the clinicopathologic features associated with shorter OS included lymphovascular invasion (*P* = 0.004), perineural invasion (*P* = 0.006), and TB-positive subgroup (*P* = 0.002) (Table 2). TB-positive subgroup was also found as a significant prognostic factor related to shorter RFS (*P* = 0.013). Other important prognostic factors for RFS included cTNM3/4 (*P* = 0.003), ypTNM3/4 (*P* < 0.001), tumor size (≥ 50 mm) (*P* = 0.023), tumor number (≥ 3) (*P* = 0.004), lymphovascular invasion (*P* < 0.001), and perineural invasion (*P* = 0.002). The multivariate analysis revealed TB-positive subgroup (HR, 2.708; 95% CI, 1.210–6.062, *P* = 0.015) were the only independent prognostic factors associated with shorter OS (Table 2), whereas ypTNM3/4 (HR, 5.439; 95% CI, 1.063–27.835, *P* = 0.042), tumor size (≥ 50 mm) (HR, 1.704; 95% CI, 1.018–2.853, *P* = 0.043), lymphovascular invasion (HR, 1.801; 95% CI, 1.027–3.156, *P* = 0.040), and TB-positive subgroup (HR, 1.748; 95% CI, 1.085–2.816, *P* = 0.022) indicated shorter RFS (Table 3).

## Discussion

A biopsy is not routinely performed for surgically resectable iCCA. However, it is a prerequisite to establish the diagnosis and facilitate subsequent NAT [42]. TB counts have been reported to be applicable in biopsy specimens, correlated well with TB counts in the following surgical resection specimens and prognostic value for survival in several other types of cancers [35, 36, 43]. A recent study [26] on treatment-naive iCCA resection specimens modified ITBCC criteria by assessing TB in whole tumor areas rather than at the invasive front because the authors believed determination of the invasive front was frequently difficult. This study also yielded a positive correlation between high TB counts and poor prognosis. These findings provide scientific and histological evidences to evaluate TB in iCCA biopsy specimens even if the tumor invasive front may not be representative on the limited biopsied tumor tissue. In our study, a relatively weak correlation between TB counts in paired pre-NAT biopsy and post-NAT resection supported the reliability of TB counts reporting in biopsy specimens (Fig. 3). The optimal prognostic cut-off value of TB counts in biopsy varies from 1 to 10 buds in the literature [43]. In our study, we have observed TB counts of 3 to be the best to predict OS, which is similar to the cut-off value in rectal adenocarcinomas [35, 36, 44]. However, such prognostic value is not well achieved in predicting RFS, possibly because not all resection cases’ biopsy slides are available to be included in the study. Further study, preferably a prospective randomized clinical trial with a larger case number, is needed to determine the best cut-off value and its prognostic power in the treatment selection of iCCA.

Recent evidence showed that a proportion of iCCA patients could benefit from NAT to downstage the iCCA in order to achieve surgical resection [20]. Pathological examination of the resection specimens delivers crucial information in the post-NAT setting in many types of cancers, whereas this topic is not well addressed in iCCA [37–40]. In our study, the AJCC tumor stages only showed suboptimal prognostic value, as the cTNM stages and ypTNM stages did not achieve statistical significance (Tables 2 and 3). The TRS also fails to demonstrate prognostic value for post-NAT iCCA patients (Supplemental Fig. 1). Therefore, additional biomarkers that are able to stratify these patients into different prognostic and potentially therapeutic groups would be highly beneficial. TB has been identified as a highly valuable histopathological parameter in a variety of cancer types [29, 30, 40, 45–47] and recent studies also demonstrated its prognostic value in treatment-naive BTCs [26–28, 40, 48]. Our study investigated TB according to the ITBCC criteria, a reproducible TB grading scheme validated by multi-center prospective studies in colon cancer, in post-NAT iCCA resection specimens and identified TB as a strong and independent prognostic factor for OS and RFS. The TB-positive group outperforms all other clinicopathological parameters, including the TNM stage, in multivariate analysis for survival analysis (Tables 2 and 3). Our finding, together with other similar TB studies in colorectal [37], esophageal [38, 49], gastric [39] cancer, and perihilar cholangiocarcinoma [40] post-NAT resection specimens, strongly supported that TB counts retained its significant prognostic power in cancer resection specimens after NAT and shall be considered to be included into the routine pathological report protocol.

Additionally, we have observed that a two-tier grading scheme, merging ITBCC intermediate and high groups into one “positive” group, showed better prognostic power than the original three-tier grading scheme in ITBCC (Fig. 4, Supplemental Fig. 3). Practically, a two-tier scheme is simpler, more cost-effective, and reproducible than a three-tier scheme in routine practices.

The statistically borderline significant, but weak positive, correlation of TB counts in biopsy and in subsequent post-NAT resection specimens (Fig. 3) suggests that TB may not only be a morphological feature of tumor growth but also related to the intrinsic aggressive biological characteristics of the tumor. As TB retained an association with poor prognosis before and after NAT, a consistent biomarker in different spatiotemporal conditions of the tumor, it may be related to certain key biological factors in driving cancer invasion and metastasis. Emerging evidences suggest that TB may play a role in epithelial-mesenchymal transition (EMT) and other molecular pathways of tumor microenvironment in promoting cancer progression [25]. Our results, in a post-NAT setting, also showed high TB counts are related to high perineural invasion. The specific pathologenetic and molecular aspects of these phenomena are worth further investigation, particularly by the latest sub-histology level analysis technology such as single cell sequencing and spatial multi-omics [50].

This study has several limitations. Patients who had undergone a biopsy but were not eligible for surgical resections were not included. And our study is a single-center retrospective cohort study in nature. A prospective multicenter study is preferred to further validate our results in the future.

## Conclusion

In summary, our study demonstrated that tumor budding, evaluated by modified ITBCC criteria, provides independent prognostic information in pre-NAT biopsy and post-NAT resection iCCA specimens. Our findings, in line with other evidences, support tumor budding to be included as a routine parameter in the pathology report of iCCA.

## Supplementary Information

Below is the link to the electronic supplementary material.

ESM 1 (PNG 159 KB)

Supplementary file2 (EPS 1985 KB)

ESM 3 (PNG 112 KB)

Supplementary file4 (EPS 1411 KB)

ESM 5 (PNG 267 KB)

High Resolution Image (TIFF 1253 kb)

Supplementary file7 (DOCX 17 KB)

Supplementary file8 (DOCX 25 KB)

## Data Availability

The data that support the findings of this study are available from the corresponding author K.Z., upon reasonable request.
