# Supplementary figures and images for: Tumor budding in pre-neoadjuvant biopsy and post-neoadjuvant resection specimens is associated with poor prognosis in intrahepatic cholangiocarcinoma—a cohort study of 147 cases by modified ITBCC criteria
Source: Virchows Arch. 2024 Oct 10;485(5):913–23. doi: 10.1007/s00428-024-03937-y (PMC11564401; doi:10.1007/s00428-024-03937-y)

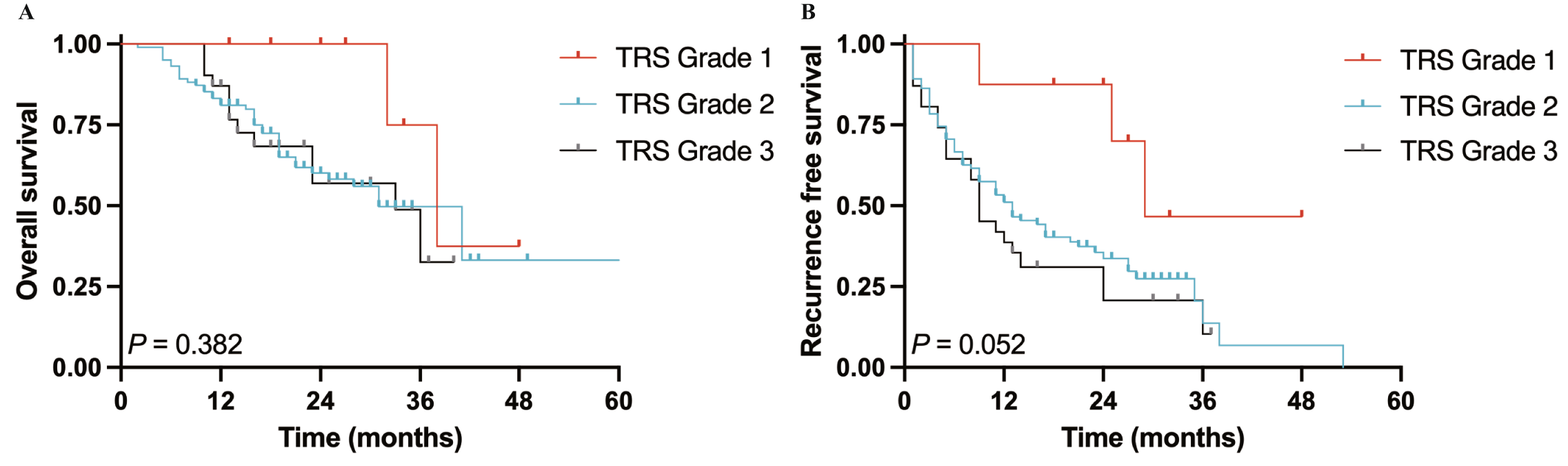

Supplement: Supplementary file 1 — (PNG 159 KB) [file 428_2024_3937_Fig6_ESM.png]

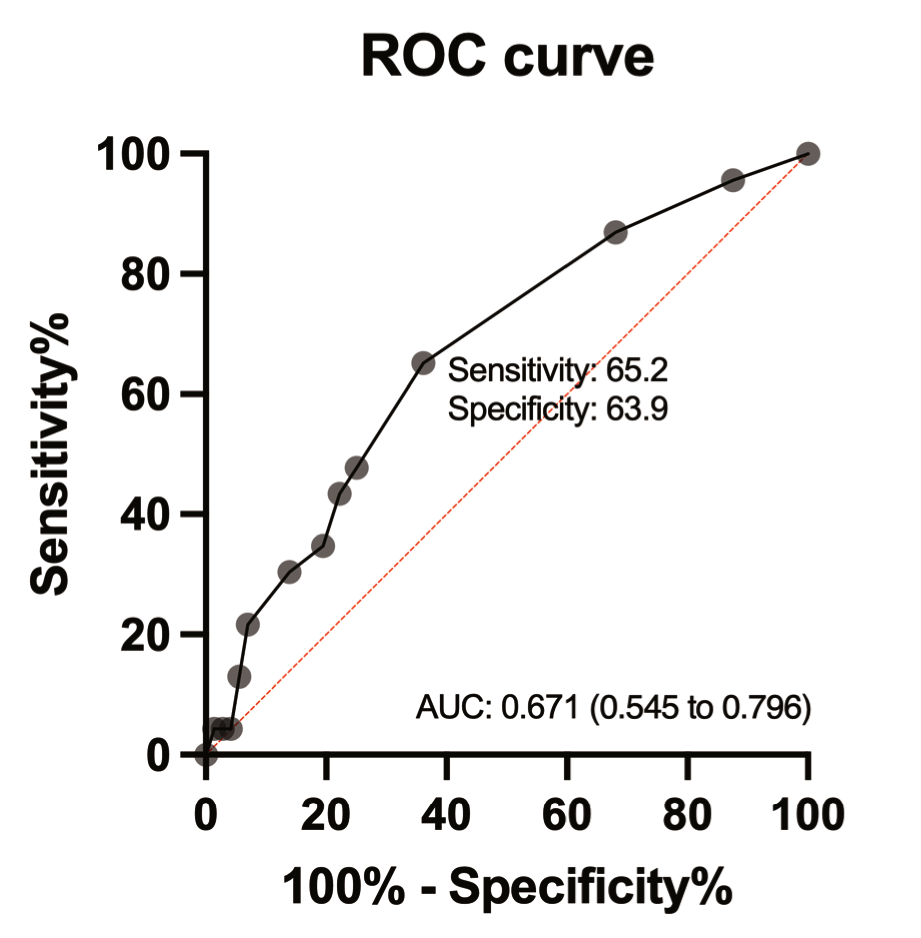

Supplement: Supplementary file 3 — (PNG 112 KB) [file 428_2024_3937_Fig7_ESM.png]

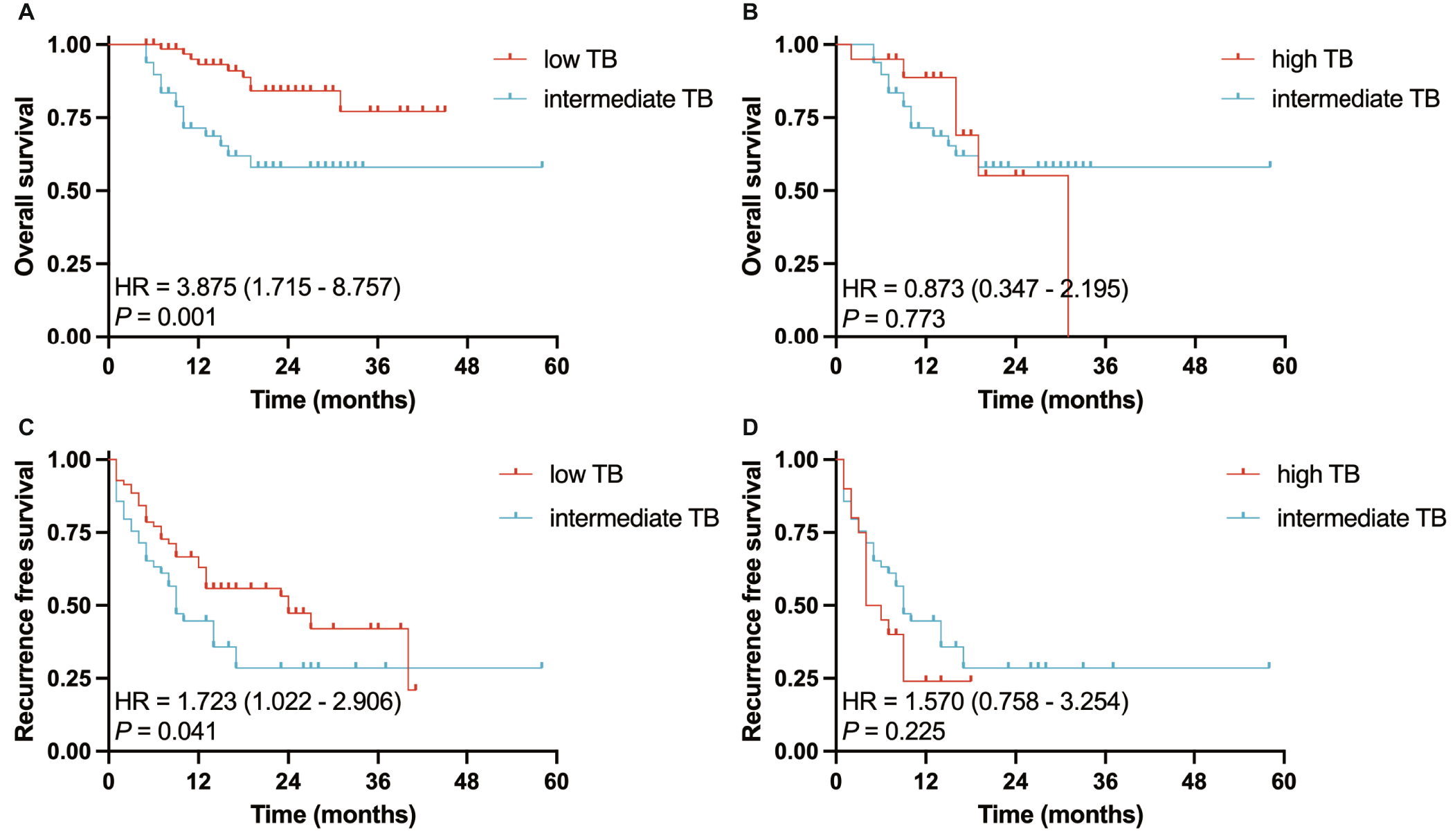

Supplement: Supplementary file 5 — (PNG 267 KB) [file 428_2024_3937_Fig8_ESM.png]
