# Supplementary material for: Tumor budding in pre-neoadjuvant biopsy and post-neoadjuvant resection specimens is associated with poor prognosis in intrahepatic cholangiocarcinoma—a cohort study of 147 cases by modified ITBCC criteria
Source: Virchows Arch. 2024 Oct 10;485(5):913–23. doi: 10.1007/s00428-024-03937-y (PMC11564401; doi:10.1007/s00428-024-03937-y)
Supplement: Supplementary file 7 — Supplementary file7 (DOCX 17 KB) [file 428_2024_3937_MOESM4_ESM.docx]

Supplemental Figure 1. Survival analysis of post-NAT iCCA using the CAP grading scheme. (A) The OS of TRS Grade 1/2/3 cases using CAP grading scheme; (B) The RFS of TRS Grade 1/2/3 cases using CAP grading scheme. NAT indicates neoadjuvant therapy; iCCA, intrahepatic cholangiocarcinoma; OS, overall survival; RFS, recurrence-free survival; CAP, the College of American Pathologists; TRS, tumor regression score.

Supplemental Figure 2. The ROC curve of TB value in pre-NAT biopsy cases. ROC indicates receiver operating characteristic; TB, tumor budding.

Supplemental Figure 3. Subgroup survival analysis of post-NAT resected iCCA using standard ITBCC criteria. A. The OS of low TB/intermediate TB cases. B. The OS of intermediate TB/high TB cases. C. The RFS of low TB/intermediate TB cases. D. The RFS of intermediate TB/high TB cases. NAT indicates neoadjuvant therapy; iCCA, intrahepatic cholangiocarcinoma; ITBCC, International Tumor Budding Consensus Conference; OS, overall survival; TB, tumor budding; RFS, recurrence-free survival.
