# Supplementary material for: Tumor budding in pre-neoadjuvant biopsy and post-neoadjuvant resection specimens is associated with poor prognosis in intrahepatic cholangiocarcinoma—a cohort study of 147 cases by modified ITBCC criteria
Source: Virchows Arch. 2024 Oct 10;485(5):913–23. doi: 10.1007/s00428-024-03937-y (PMC11564401; doi:10.1007/s00428-024-03937-y)
Supplement: Supplementary file 8 — Supplementary file8 (DOCX 25 KB) [file 428_2024_3937_MOESM5_ESM.docx]

| Supplemental Table 1. Associations Between Tumor Budding and Clinicopathologic Features of 95 pre-NAT biopsy iCCA Patients | | | |
| --- | --- | --- | --- |
|  | **Biopsy** | | |
| **Parameter** | **TB-negative (%)** | **TB-positive (%)** | **P** |
| Age |  |  | 0.474 |
| <65 | 38(70.4) | 26(63.4) |  |
| ≥65 | 16(29.6) | 15(36.6) |  |
| Gender |  |  | 0.843 |
| Male | 34(63.0) | 25(61.0) |  |
| Female | 20(37.0) | 16(39.0) |  |
| HBV infection |  |  | 0.817 |
| Negative | 37(68.5) | 29(70.7) |  |
| Positive | 17(31.5) | 12(29.3) |  |
| CEA (ng/mL) |  |  | 0.765 |
| <5 | 46(85.2) | 34(82.9) |  |
| ≥5 | 8(14.8) | 7(17.1) |  |
| CA19-9 (U/mL) |  |  | 0.006* |
| <37 | 49(76.6) | 15(48.4) |  |
| ≥37 | 15(23.4) | 16(51.6) |  |
| cTNM |  |  | 0.718 |
| I | 5(9.3) | 6(14.6) |  |
| II | 3(5.6) | 3(7.3) |  |
| III | 41(75.9) | 30(73.2) |  |
| IV | 5(9.3) | 2(4.9) |  |
| Tumor size (median = 50) (mm) |  |  | 0.602 |
| <50 | 17(31.5) | 15(36.6) |  |
| ≥50 | 37(68.5) | 26(63.4) |  |
| Tumor number |  |  | 0.159 |
| 1 | 36(66.7) | 33(80.5) |  |
| 2 | 4(7.4) | 1(2.4) |  |
| ≥3 | 14(25.9) | 7(17.1) |  |
| Vascular invasion |  |  | 0.335 |
| Negative | 33(61.1) | 21(51.2) |  |
| Positive | 21(38.9) | 20(48.8) |  |

NAT indicates neoadjuvant therapy; iCCA, intrahepatic cholangiocarcinoma; TB, tumor budding; HBV, hepatitis B virus.

| Supplemental Table 2. Univariate and Multivariate Analyses of Factors Associated with Overall Survival of 95 biopsy pre-NAT iCCA Patients | | | | | |
| --- | --- | --- | --- | --- | --- |
|  | **Univariate** | |  | **Multivariate** | |
| **Parameter** | **HR** | **P** |  | **HR** | **P** |
| Age (≥65) | 1.444(0.624-3.340) | 0.390 |  |  |  |
| Gender (male) | 1.510(0.615-3.707) | 0.369 |  |  |  |
| HBV infection status (positive) | 0.947(0.591-1.517) | 0.821 |  |  |  |
| CEA (≥5 ng/mL) | 2.226(0.814-6.091) | 0.119 |  |  |  |
| CA19-9 (≥37 U/mL) | 2.011(0.858-4.716) | 0.108 |  |  |  |
| cTNM3/4 | 1.511(0.448-5.093) | 0.506 |  |  |  |
| Tumor size (≥50 mm) | 1.435(0.608-3.389) | 0.410 |  |  |  |
| Tumor number (≥3) | 2.417(1.038-5.626) | 0.041* |  | 2.510(1.042-6.046) | 0.040* |
| Vascular invasion | 1.027(0.448-2.356) | 0.950 |  |  |  |
| TB subgroup (TB-positive) | 3.105(1.304-7.390) | 0.010* |  | 2.806(1.113-7.077) | 0.029* |

NAT indicates neoadjuvant therapy; iCCA, intrahepatic cholangiocarcinoma; HBV, hepatitis B virus; TB, tumor budding.

| Supplemental Table 3. Univariate and Multivariate Analyses of Factors Associated with Recurrence-Free Survival of 95 pre-NAT biopsy iCCA Patients | | | | | |
| --- | --- | --- | --- | --- | --- |
|  | **Univariate** | |  | **Multivariate** | |
| **Parameter** | **HR** | ***P*** |  | **HR** | ***P*** |
| Age (≥65) | 0.502(0.256-0.987) | 0.100 |  |  |  |
| Gender (male) | 1.696(0.919-3.132) | 0.091 |  |  |  |
| HBV infection status (positive) | 1.466(0.803-2.678) | 0.213 |  |  |  |
| CEA (≥5 ng/mL) | 1.241(0.580-2.655) | 0.577 |  |  |  |
| CA19-9 (≥37 U/mL) | 1.501(0.824-2.734) | 0.185 |  |  |  |
| cTNM3/4 | 3.372(1.207-9.418) | 0.020* |  | 1.854(0.504-6.817) | 0.353 |
| Tumor size (≥50 mm) | 1.696(0.938-3.067) | 0.081 |  |  |  |
| Tumor number (≥3) | 2.309(1.194-4.465) | 0.013* |  | 2.310(1.158-4.607) | 0.018* |
| Vascular invasion | 0.643(0.088-4.671) | 0.662 |  |  |  |
| TB subgroup (TB-positive) | 1.456(0.825-2.568) | 0.194 |  |  |  |

NAT indicates neoadjuvant therapy; iCCA, intrahepatic cholangiocarcinoma; HBV, hepatitis B virus; TB, tumor budding.
